# Exploring Faculty Preparedness for Artificial Intelligence-Driven Dental Education: A Multicentre Study

**DOI:** 10.7759/cureus.64377

**Published:** 2024-07-11

**Authors:** Saad M Al-Zubaidi, Gul Muhammad Shaikh, Asma Malik, Malik Zain Ul Abideen, Jawad Tareen, Nada Saeed A Alzahrani, Ammar Ahmed Siddiqui

**Affiliations:** 1 Department of Restorative Dental Sciences, College of Dentistry, University of Hail, Hail, SAU; 2 Department of Dental Education, Shahida Islam Medical and Dental College, Lodhran, PAK; 3 Department of Medical Education, Foundation University School of Health Sciences, Islamabad, PAK; 4 Department of Dental Education and Research, Bakhtawar Amin Medical and Dental College, Multan, PAK; 5 Department of Medical Education, Bakhtawar Amin Medical and Dental College, Multan, PAK; 6 Hail Specialised Dental Centre, Ministry of Health, Hail, SAU; 7 Department of Preventive Dental Sciences, College of Dentistry, University of Hail, Hail, SAU

**Keywords:** qualitative research, faculty, dental, dental education, curriculum, artificial intelligence

## Abstract

Introduction

In the modern era, technology, including artificial intelligence (AI), is the centre of digital innovation. AI is revolutionising numerous fields, including the healthcare sector, globally. Incorporating AI in dental education may help in improving the diagnostic accuracy, learners’ experiences, and effectiveness of the management of dental education institutions. However, successful implementation of AI requires the faculty's willingness to incorporate it into their practices. Thus, this research aims to explore the readiness of faculty members to integrate AI into dental education.

Methodology

The study employed a qualitative exploratory design to gather in-depth insights into faculty readiness for AI-driven dental education. Purposive sampling was employed, and 21 faculty members from public and private dental colleges in South Punjab participated in semi-structured interviews. The interviews focused on understanding participants' perceptions, experiences, and challenges related to AI integration in dental education. Thematic analysis was conducted utilising Braun and Clarke's framework to identify key themes and subthemes from the qualitative data using inductive coding.

Results

Five major themes and 14 subthemes emerged from the data analysis. Faculty members had low AI literacy coupled with diverse perceptions; some participants perceived AI as a solution for revolutionising teaching and learning, while others criticised its misuse as academic misconduct by students, an effect on students’ critical thinking, and a threat to conventional jobs. However, most of the respondents also considered AI beneficial for students with remote access or from marginalised populations in terms of accessing and learning from limited resources. Concerns that participants highlighted included a lack of training opportunities, limited facilities, ethical concerns pertaining to data privacy, and assessment bias. Some of the recommendations provided by the respondents include the provision of training opportunities, the allocation of resources and infrastructure, and continuous effective support from institutions for the integration of AI in dental education.

Conclusions

This study emphasised the readiness of the faculty when it comes to the integration of AI in dental education. The faculty considered AI favourable for digitization and innovative education, although there is a lack of awareness of its application. Regarding the benefits of utilising AI, respondents highlighted its quick response, prediction of students’ performance, and flexibility in learning. The challenges included lack of awareness regarding its implementation, inadequate training, lack of availability of resources, lack of institutional support, the problem of data confidentiality, and resistance to change. Suggestions included the provision of technical support, skills training, and the provision of required infrastructure. Participants recommended that AI tools must incorporate cultural and contextually specific content, use technical support for problems, and incorporate constant response systems to improve the AI tools for novice users, especially within developing regions such as Pakistan.

## Introduction

In the current era of technological revolution, the world is evolving rapidly, and artificial intelligence (AI) is at the forefront of this advancement. AI is the ability to understand and mimic human intelligence through machine learning, neural networks, and natural language processing [1].

AI is now extensively utilised in educational and health systems, where the dissemination of knowledge and provision of patient care is being enhanced. In the field of dentistry, AI is enhancing the analysis of diagnostics, disease control, and treatment, as well as patient-personalised care. In the sector of education, it is changing approaches to teaching and learning [2]. AI can reduce assessment bias, provide timely feedback, improve students’ knowledge base and skills, and enable a safe learning experience and personalised learning modes, especially during preclinical and clinical phases [3]. Moreover, AI saves time for faculty by helping to recognise students who require special attention in academics, thus sparing more time for valuable academic activities. The World Health Organization (WHO) urged healthcare practitioners to learn the basic operations of AI and to contribute to making AI an essential component of healthcare learning [4]. This is particularly important because inadequate implementation of AI can result in wrong diagnoses and poor treatment planning [4,5]. While most of the practitioners are not only unfamiliar with fundamental AI science, concepts, or ethics, they only use AI in basic applications like voice and facial recognition. Beyond the current applications, AI has the potential to further transform dental education in various ways. Future explorations could include the development of personalised learning plans tailored to individual student needs and progress, advanced simulation-based training using virtual reality (VR) and augmented reality (AR), and the use of AI for predictive analytics to identify students at risk of underperformance. Additionally, AI can facilitate remote learning and tele-mentoring, expanding access to quality dental education, particularly in underserved regions [4]. Although AI offers numerous benefits, it also raises ethical concerns that need to be addressed. These include data privacy and security, given the sensitive personal data AI systems require. There are also concerns about algorithmic bias, which can affect fairness and decision-making. Ethical considerations further extend to the transparency and accountability of AI systems and ensuring informed consent from data subjects. Addressing these ethical challenges is crucial for the responsible integration of AI in dental education [5].

A review of the findings has also shown that there is a lack of relevant knowledge among the healthcare professions as far as AI is concerned, affecting its implementation. Teaching faculty needs to have a minimum level of AI proficiency for its effective utilisation [6]. In the near future, healthcare workers who are conversant with AI will replace practitioners lacking such knowledge due to the rapid takeover of AI. Therefore, strategic planning is required to integrate and implement AI in healthcare education. AI has a vast future in many fields, but especially in the medical and dental fields. Hence, training the trainers regarding AI is the need of the hour [7].

Incorporation of AI in healthcare requires infrastructure investments, workforce development, and the formulation of regulations. However, it is very important to explore the faculty readiness for this revolutionary digital transformation; readiness is an important aspect in determining efficiency in the adoption and implementation of AI technologies in the teaching and learning of dental subjects [8]. AI preparedness in healthcare, which includes components of knowledge, attitude, and skills regarding AI technologies and applications, is quite different from AI literacy. AI preparedness has been studied in the context of healthcare, engineering, business development, and governmental initiatives in different notable countries such as China [9].

As the global economy gradually adapts to AI advancements in healthcare, the healthcare sector in Pakistan is also beginning to pay attention to the utilisation of AI, which will result in optimal utilisation of resources and improved diagnostic accuracy. Therefore, it is very important to train future healthcare professionals by integrating AI into dental education [10]. However, a key factor is the preparedness and willingness of institutions and faculty to incorporate AI into the teaching process to make it successful [9].

Several reviews and commentaries have explored the medical applications of AI, while a limited number of original papers have examined in depth the faculty preparedness towards AI among faculty members of dental schools [11]. It is noteworthy that many of these studies focused on AI's use in radiology. Additionally, a few studies have investigated the perceptions, awareness, and knowledge of medical and dental faculty and students in Pakistan regarding AI [12,13].

Despite the growing recognition of AI's importance in healthcare, to the best of our knowledge, no study explored the preparedness of faculty members to integrate AI into dental education, particularly in South Punjab, Pakistan, a developing country with limited resources. This study aims to explore faculty preparedness regarding AI in dental education. By exploring the current state of faculty readiness, this study aims to facilitate the integration of AI into dental education in South Punjab.

## Materials and methods

The study utilised a qualitative exploratory design to explore the experiences and readiness of dental faculty regarding AI-driven education in dental institutions. The targeted population was comprised of faculty members (assistant professors and above) from Pakistan Medical and Dental Council (PMDC)-recognised dental institutes in South Punjab. The total study population was 76 faculty members. A total of 21 faculty members from dental schools across South Punjab, Pakistan were recruited through non-probability purposive sampling, ensuring a diverse representation of specialities and teaching experiences. South Punjab has a total of five dental institutions: four private and one public. Faculty members were recruited from all these colleges to ensure diverse perspectives. Specifically, 13 faculty members were recruited from the private colleges (with a breakdown of three, three, three, and four from each of the four private colleges), and eight faculty members were recruited from the public college. The sampling process was carried out until data saturation was reached, indicating that no new ideas or themes were reported from interviews.

Faculty members included in the study had faculty registration with the PMDC, held the rank of assistant professor or above, were willing to participate, and provided informed consent. Faculty members excluded from the study were those without PMDC registration, below the rank of assistant professor, or who declined to participate or did not provide informed consent.

A written informed consent was obtained in person from all participants prior to the interview, ensuring data safety and allowing participants to withdraw from the study at any time. An information sheet was also attached along with informed consent outlining the study's objective, methodology, potential benefits, and hazards. The data were acquired through semi-structured interviews with open-ended questions about areas like how people understand and feel about AI, what they know about and what they have done with AI tools now, what they think are the pros and cons of AI, their training needs and preferences, the support and infrastructure at their institution, and ethical issues. The semi-structured interview guide was prepared from an extensive literature search. The data were collected through semi-structured interviews based on the objectives of the research and modified further using expert validation and pilot testing of the content for clarity, relevance, and cultural appropriateness. The interview guide was revised and modified based on expert feedback and pilot testing. The timing of the semi-structured interviews ranged from 23 to 37 minutes, which allowed for the collection of adequate information. In conducting interviews, face-to-face sessions were preferred because most of the participants agreed to participate in this type of interaction. Consent was obtained from the participants to record these interviews in audio format. Field notes were also taken to record vital contextual features such as facial gestures, emotions, and minute changes in the reactions of the participants.

Ethical permission for the study was obtained from the Institutional Review Board of Bakhtawar Amin Medical and Dental College (approval number: COD 319/23).

The transcripts were transcribed through the software Otter.ai (Otter.ai, Inc., Mountain View, CA), and coding was performed manually. Data analysis followed an inductive thematic approach utilising the Braun and Clark thematic analysis framework [14].

**Figure 1:**
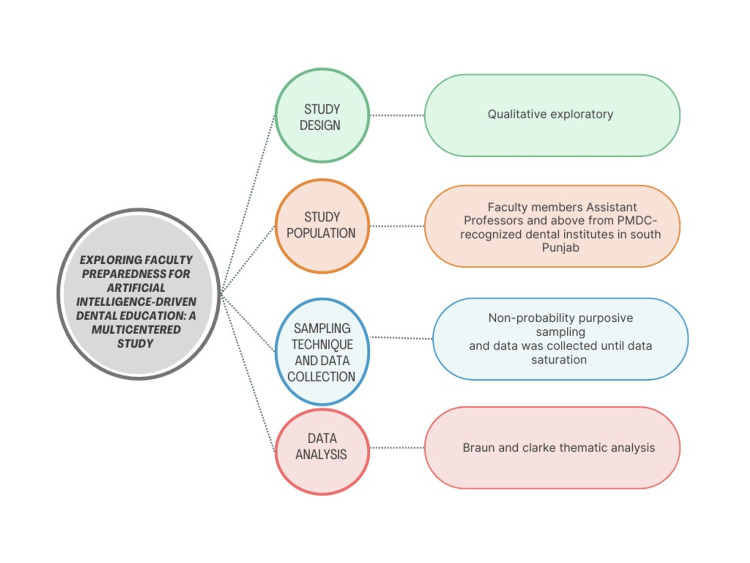
Methodology flowchart. PMDC: Pakistan Medical and Dental Council. Media element by Canvalisa via canva.com (Canva).

Preliminary transcripts were shared with the participants through email and WhatsApp for member checking. The confidentiality of the participants was maintained by anonymizing all the collected data. The coding type employed in the analysis was primarily inductive, allowing themes and patterns to emerge directly from the data, supplemented by open coding techniques to ensure a thorough exploration of the dataset. Tables were utilised to manage the large volume of gathered information. Two researchers meticulously transcribed the audio tape recordings and field notes into text data, which were then cross-checked by the principal investigator to ensure quality. Similar data points were grouped to form major ideas or themes within the database. The analysis continued until no new information or themes emerged (Figure 1).

## Results

Table 1 presents the demographic details of the 21 faculty members who participated in the study. The sample comprised 11 male respondents (52.4%) and 10 females (47.6%). Participants' ages were distributed across three ranges: 30-39 years (six participants, 28.6%), 40-49 years (eight participants, 38.1%), and 50-59 years (seven participants, 33.3%). In terms of academic positions, seven participants were professors (33.3%), eight were associate professors (38.1%), and six were assistant professors (28.6%). Regarding teaching experience, three had one to five years (14.3%) of experience, six had six to 10 years (28.6%), seven had 11-15 years (33.3%), three had 16-20 years (14.3%), and two had 21-25 years (9.5%) of experience. The faculty members were from different specialized fields, including paediatric dentistry (4.8%), orthodontics (9.5%), prosthodontics (9.5%), oral surgery (9.5%), endodontics (4.8%), periodontology (9.5%), operative dentistry (9.5%), oral pathology (9.5%), dental materials (9.5%), community dentistry (9.5%), and oral biology (9.5%). The participants were from both public (13 participants, 61.9%) and private (eight participants, 38.1%) dental schools (Table 1).

**Table 1:** Demographic characteristics of the participants.

Characteristics	Frequency (n)	Percentage (%)
Gender		
Male	11	52.4
Female	10	47.6
Age range (years)		
30-39	6	28.6
40-49	8	38.1
50-59	7	33.3
Academic position		
Professor	7	33.3
Associate professor	8	38.1
Assistant professor	6	28.6
Teaching experience (years)		
1-5 years	3	14.3
6-10 years	6	28.6
11-15 years	7	33.3
16-20 years	3	14.3
21-25 years	2	9.5
Specialization		
Paediatric dentistry	2	9.5
Orthodontics	2	9.5
Prosthodontics	2	9.5
Oral surgery	2	9.5
Endodontics	1	4.8
Periodontology	2	9.5
Operative dentistry	2	9.5
Oral pathology	2	9.5
Dental materials	2	9.5
Community dentistry	2	9.5
Oral biology	2	9.5
Institution type		
Government/public dental school	8	38.1
Private dental school	13	61.9

**Figure 2:**
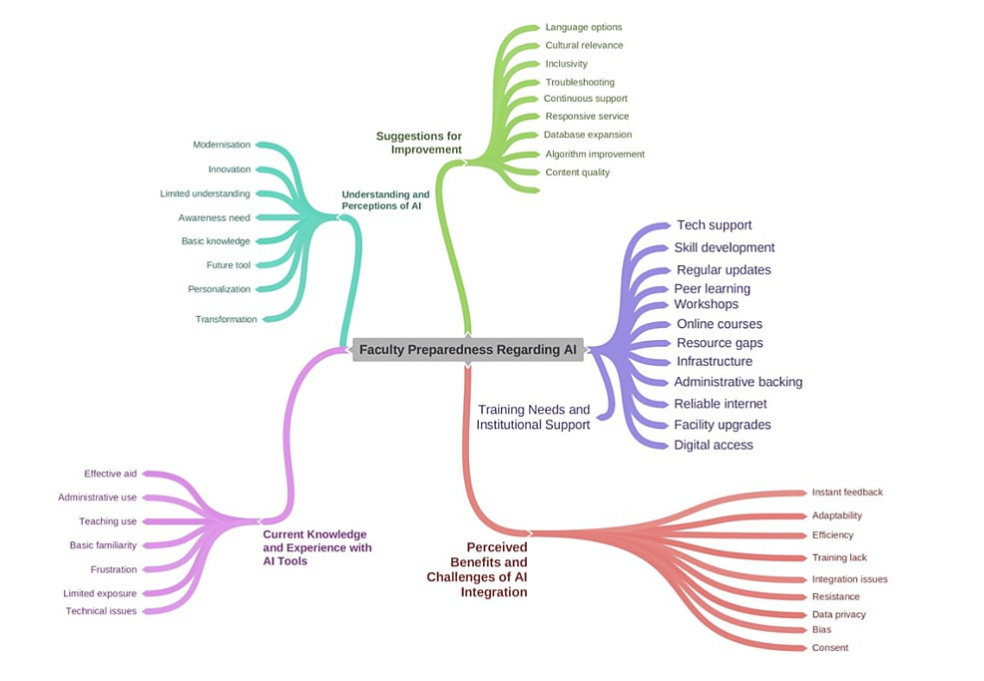
Qualitative results. AI: artificial intelligence. Media element by coggle.it (Coggle).

Multidimensional insights into the faculty readiness for AI-driven education in Pakistani medical and dentistry schools were obtained from the thematic analysis of qualitative data (Figure 2).

Participants exhibited varying levels of awareness and understanding of AI, with some (24%) viewing it as a transformative tool for education while others expressed apprehension about its potential to disrupt traditional teaching methods and affect their conventional jobs. While participants held positive attitudes towards AI's potential benefits, they also expressed concerns over perceived difficulties like the lack of provision of basic facilities like the internet and infrastructure in a developing country like Pakistan.

The majority of the faculty members (62%) had low awareness of AI tools related to teaching and learning. A few members used some AI tools for presentation and administrative purposes. The study showed that participants had varying levels of exposure to AI tools, with some expressing interest in learning more about their potential applications in education. Respondents also pointed out the limitations of AI tools in performing specific and contextualized tasks.

Perceived benefits of AI included its effectiveness and capability to promulgate learner-centred elements such as individualised learning and performance feedback in real time. Participants described anticipated challenges pertaining to the implementation of AI, including inadequate training, a lack of technical support, a lack of infrastructure, and ethical concerns related to data privacy and results bias.

Most participants (57%) emphasised that comprehensive practical training is essential to keeping up with digital students of the era. Faculty members demonstrated interest in hands-on workshops and peer-learning sessions to apply AI in classroom and clinical sessions. Participants also emphasised the formulation of a modified mission and vision in line with the current developments of AI in healthcare. The formulation of policy and guidelines from regulators (PMDC) can play a vital role in the allocation of resources for AI by institutions.

Participants (48%) suggested that providing multilingual support and dedicated technical assistance would enhance the accessibility and usability of AI tools. Expanding the database and improving the algorithm of AI tools to generate more accurate and contextually relevant responses were deemed crucial for their effective use in education (Table 2).

**Table 2:** Faculty perspectives on AI in dental education. AI: artificial intelligence; PMDC: Pakistan Medical and Dental Council.

Themes	Subthemes	Representative quotations
Understanding and perceptions of AI	Understanding of AI	"AI is the future of medical and dental education, but many of us still have a limited understanding of its full potential." (M45PR4)
	Perception of AI’s role	"AI can significantly enhance learning, making education more interactive and personalized for students and may also compromised our conventional roles as facilitators jeopardizing our jobs." (F48GR12)
Current knowledge and experience with AI tools	Familiarity with AI tools	"We are not much familiar with advanced AI tools. I have used some free AI tools for tasks like presentation preparation and administrative work, but not extensively in my teaching. I believe AI can offer some helpful templates, but it is essential for us to focus on enhancing the content and quality of our PowerPoint presentations."(M35PR07)
	Specific experiences with AI	“I have mixed experience with AI. It can be a great aid by providing creative ideas, but it is superior for general tasks. Currently, generative AI lacks specificity and does not provide quality content based on contextualization.”(F32PR18)
Perceived benefits and challenges of AI integration	Benefits of AI integration	"AI can personalize learning and provide instant feedback, which is immensely beneficial for students. Moreover, it can facilitate students from peripheries and remote areas with limited resources." (M46GR05)
	Challenges in adoption	"The main challenge is the lack of training and infrastructure to support AI technologies in our institutions. Moreover, lack of flexibility to adopt to evolving digital landscape in teaching and learning." (M39GR19)
	Ethical concerns	"There are ethical issues, like data privacy and the potential for AI to reinforce biases, that need careful consideration. Moreover, academic integrity can be compromised and students critical thinking abilities will be compromised with earlier exposure to these tools." (F43PR14)
Training needs and institutional support	Training requirements	"We need comprehensive training programs to understand how to effectively use AI in our teaching." (M39PR09)
	Preferred training methods	"I prefer hands-on workshops from AI experts and peer learning sessions to gain practical skills in AI." (F47GR11)
	Institutional support	"There are no limitations for AI use in our institutions, but there is a gap in vision and mission for adopting AI and curriculum by providing the necessary resources and infrastructure.”(M33PR20)
	Infrastructure needs	"Reliable internet, access to advanced AI tools, and allocation of space like the formulation of AI labs are critical for successful implementation. PMDC intervention in this regard could be beneficial and will be followed by all the institutions.” (M34PR21)
Suggestions for improvement	Multilingual support	"Providing AI tools that support multiple languages would make them more accessible and useful for our diverse student population." (F51GR15)
	Technical assistance	"Having dedicated technical support to troubleshoot AI-related issues would be very helpful for beginners." (M48P13)
	Enhanced content generation	"Expanding the database and improving the algorithm of AI tools to generate more accurate and contextually relevant responses are crucial." (M32PR03)

## Discussion

The study aimed to explore the preparedness of the faculty members towards the implementation of AI in dental education in South Punjab, Pakistan. The findings revealed varying awareness and varied experiences of faculty towards AI. Participants also shed light on the perceived benefits and challenges of the implementation of AI.

Although faculty members were aware of AI's potential to revolutionise education, they were unsure about the practical implementation due to a lack of awareness and use of AI. Few participants considered these a threat to academic integrity and the traditional teaching and learning system. The findings corroborate another study conducted in 2021, which stated the lack of knowledge about AI technology, associated with a fear of being replaced by AI, was a reason for the resistance to AI implementation [15]. The findings are also in line with the narrative review published in 2023 by Morandini et al., stating that organizations like businesses, the finance sector, and healthcare are currently looking and opting for AI options and preferring individuals with expertise in AI [16].

Respondents mainly used AI for creating presentations and other low-impact administrative tasks rather than for educational purposes, leading to limited knowledge of its application in teaching and learning. This highlights the importance of developing integration strategies and providing adequate support through training and adequate facilities. The findings of the study supported another review published in 2023 by Malerbi et al., that emphasised the necessity of faculty training to bridge the knowledge gap in AI applications [17].

The study highlighted the perceived benefits of AI, including instant and customised feedback to students even in remote areas where facilitators are not available, and also facilitating and helping faculty make more effective and efficient use of time. The findings are also in line with another review published in 2023 by Dave et al., stating AI can complement and boost human tasks by facilitating students and faculty in remote areas with limited human resources, thus having a far-reaching impact in academia and healthcare [18].

The challenges identified include inadequate training, insufficient infrastructure, ethical concerns, assessment bias, academic misconduct, and effects on critical thinking abilities. A previous review published in 2023 by Srivastava et al. also highlighted ethical concerns, particularly around data privacy and bias, underscoring the need for ethical guidelines and frameworks [19].

Most of the faculty members expressed the need for training, specifically hands-on workshops and peer learning sessions to develop practical AI skills. This finding aligns with existing literature that highlights the importance of faculty training in effective AI integration [20]. Despite general institutional support for AI, the gap in resources and infrastructure poses a significant challenge, indicating a need for strategic planning and investment to support AI adoption in education.

Some of the recommendations included adding support for multiple languages, providing dedicated support technicians, and enabling the creation of specific content. These suggestions emphasise the importance of ensuring that AI tools are compatible with relevant interfaces for all users, particularly in diverse educational settings like that in Pakistan. This is also backed by the literature, which emphasises designing AI tools tailored to specific contexts for seamless integration across various disciplines [21].

Respondents claimed a lack of practical training and the importance of the institutions’ support were the factors predicting a successful AI implementation. Participants identified similar challenges related to ethical issues as those reported by another study conducted by Standish et al. in 2022 in the US [22]. The findings suggest that these barriers are universal across different contexts [23].

Limitations

The limitations of the study included the possibility of response bias due to self-reported data provided by participants and the respondents could have given answers that are socially acceptable. Additionally, limiting the study to South Punjab restricts the applicability of findings to other regions. Moreover, although it is very detailed, the qualitative method makes it difficult to quantify how prepared faculty members are for AI integration. To validate and build upon these results, future studies should use larger and more diverse samples employing mixed method design, and include the viewpoints of students, administrators, and policymakers to give a thorough picture of institutional readiness for AI in dental education.

## Conclusions

The study highlighted the significance of faculty preparedness regarding the integration of AI in dental education. The pros of AI integration include enhanced digitization, innovation, and various educational benefits like self-developed learning content based on the learner’s strengths and potential dialogues to improve their communication skills. However, there are significant cons that need addressing. These include concerns related to autonomy, human welfare, safety, public interest, openness, transparency, explainability, responsibility, accountability, data governance, inclusion, and fairness. Faculty members considered AI a beneficial aid to digitization and innovation; however, the majority were unaware of its implementation. Most of the faculty members highlighted the advantages of AI, including instant and customised feedback, predictive student performance indications, and flexible learning. While key challenges included lack of knowledge and training of faculty, integration issues due to limited resources and lack of institutional support, problems of data privacy, and the reluctance of staff to adopt changes, institutions need to address these gaps by providing technical support, skill development, and digital access to faculty and students. Moreover, providing cultural relevance, troubleshooting, and a continuous response system for AI software can help improve its implementation in developing countries like Pakistan.

## Appendices

**Table 3:** Semi-structured interview guide for faculty members.

#	Interview guide questions
1.	Can you describe your understanding of artificial intelligence (AI) in the context of dental education?
2.	How do you perceive the role of AI in transforming dental education?
3.	What AI tools or technologies are you familiar with or have used in your teaching practice?
4.	Can you share any specific experiences with these tools?
5.	What do you see as the primary benefits of integrating AI into dental education?
6.	What challenges or barriers do you anticipate or have already encountered in adopting AI technologies in your teaching?
7.	What ethical considerations or concerns do you have about the use of AI in dental education?
8.	How do you think institutions should address these issues?
9.	What specific training or professional development do you feel you need to effectively incorporate AI into your teaching?
10.	How would you describe the level of institutional support for integrating AI into the curriculum at your institution?
11.	Do you have any additional thoughts or comments on the topic of AI in dental education that we have not covered?
